# Sprint Specificity of Isolated Hamstring-Strengthening Exercises in Terms of Muscle Activity and Force Production

**DOI:** 10.3389/fspor.2020.609636

**Published:** 2021-01-21

**Authors:** Caroline Prince, Jean-Benoît Morin, Jurdan Mendiguchia, Johan Lahti, Kenny Guex, Pascal Edouard, Pierre Samozino

**Affiliations:** ^1^University of Savoie Mont Blanc, Laboratoire Interuniversitaire de Biologie de la Motricité (EA7424), Chambéry, France; ^2^Physiotherapy and Sports Medicine Department, Swiss Olympic Medical Center, La Tour Hospital, Meyrin, Switzerland; ^3^University of Lyon, UJM-Saint-Etienne, Inter-university Laboratory of Human Movement Science (LIBM EA 7424), Saint-Étienne, France; ^4^Sports Performance Research Institute New Zealand, School of Sport and Recreation, Auckland University of Technology, Auckland, New Zealand; ^5^Department of Physical Therapy, ZENTRUM Rehab and Performance Center, Barañain, Spain; ^6^University of Côte d'Azur, LAMHESS, Nice, France; ^7^School of Health Sciences (HESAV), University of Applied Sciences and Arts Western Switzerland (HES-SO), Lausanne, Switzerland; ^8^Head Coach Sprint/Hurdles/Relays Coach 400m/400m Hurdles Swiss Athletics, Haus des Sports, Ittigen, Switzerland; ^9^Sports Medicine Unit, Department of Clinical and Exercise Physiology, Faculty of Medicine, Regional Institute of Medicine and Sports Engineering (IRMIS), University Hospital of Saint-Etienne, Saint-Ètienne, France

**Keywords:** track and field, hamstring muscles, sprint mechanics, strength exercises, horizontal force production

## Abstract

To train hamstring muscle specifically to sprint, strengthening programs should target exercises associated with horizontal force production and high levels of hamstring activity. Therefore, the objectives of this study were to analyze the correlation between force production capacities during sprinting and hamstring strengthening exercises, and to compare hamstring muscle activity during sprinting and these exercises. Fourteen track and field regional level athletes performed two maximal 50-m sprints and six strengthening exercises: Nordic hamstring exercises without and with hip flexion, Upright-hip-extension in isometric and concentric modalities, Standing kick, and Slide-leg-bridge. The sprinting horizontal force production capacity at low (F0) and high (V0) speeds was computed from running velocity data. Hamstring muscle performances were assessed directly or indirectly during isolated exercises. Hamstring muscle electromyographic activity was recorded during all tasks. Our results demonstrate substantially large to very large correlations between V0 and performances in the Upright-hip-extension in isometric (r_s_ = 0.56; *p* = 0.040), Nordic hamstring exercise without hip flexion (r_s_ = 0.66; *p* = 0.012) and with 90° hip flexion (r_s_ = 0.73; *p* = 0.003), and between F0 and Upright-hip-extension in isometric (r_s_ = 0.60; *p* = 0.028) and the Nordic hamstring exercise without hip flexion (r_s_ = 0.59; *p* = 0.030). However, none of the test exercises activated hamstring muscles more than an average of 60% of the maximal activation during top-speed sprinting. In conclusion, training programs aiming to be sprint-specific in terms of horizontal force production could include exercises such as the Upright-hip-extension and the Nordic hamstring exercise, in addition to maximal sprinting activity, which is the only exercise leading to high levels of hamstring muscle activity.

## Introduction

Sprinting acceleration is a key component in numerous sports aiming to cover a given distance in the shortest time (Morin et al., 2011; Bourne et al., 2018). Sprint acceleration performance is known to be influenced by sprinters' capabilities to produce and maintain backward horizontally oriented force onto the ground (Morin et al., 2011, 2012). Hip extensor muscles (hamstring and gluteal muscles) appear to be the main contributors to such oriented force. More specifically, it appears that the ability of the hamstring muscles to produce horizontally oriented force during the stance phase is determined by the hamstring muscles' ability to produce high levels of eccentric force and to be activated at the end of the swing phase (Morin et al., 2015; Edouard et al., 2018). Hamstring muscles' role vary through the sprint. From a neuromuscular perspective, most of their activity is generated from the late swing to the end of the stance phase (Yu et al., 2008; Howard et al., 2018). From a kinematic perspective, the biceps femoris and semitendinosus muscles reach their greatest length in the late swing phase (Yu et al., 2008; Schache et al., 2012). Due to the fact that the hip joint reaches peak flexion while the knee joint begins to extend (Kenneally-Dabrowski et al., 2019), strain stress on the hamstring increase in this phase (Guex and Millet, 2013). Additionally, force production peaks during this phase (Schache et al., 2012). Thus, hamstring muscles play a major role in high-speed running (Morin et al., 2015), and sprinting solicits hamstring muscles in various modalities, more specifically from the mid-swing phase to the beginning of the stance phase (Yu et al., 2008; Schache et al., 2012; Howard et al., 2018; Kenneally-Dabrowski et al., 2019). These specificities should be taken into account when selecting isolated training exercises that aim to stimulate and train hamstring function with the highest possible degree of transfer to actual sprint tasks and performance.

In addition to their important role in sprint mechanics and performance (Schache et al., 2012; Morin et al., 2015), hamstring muscles appear to be the most vulnerable muscles in sports involving high-speed running (Edouard et al., 2016; Ekstrand et al., 2016). Therefore, hamstring-strengthening exercises have been proposed as a promising approach in the primary prevention of hamstring muscle injury (HMI) (Al Attar et al., 2017; Bourne et al., 2018), and their efficacy to decrease HMI has been discussed in the context of interventional studies (Gabbe et al., 2006; Goode et al., 2015; Al Attar et al., 2017; van Dyk et al., 2019). However, HMI remains a major injury in sprint-related sports (Edouard et al., 2016; Ekstrand et al., 2016). One explanation could be the lack of sprint-specificity of hamstring-strengthening exercises (Malliaropoulos et al., 2012; Guex and Millet, 2013; van den Tillaar et al., 2017), which could limit the potential benefits of strength gain transfer from exercises to force production or resisting muscle strain stress during sprint. van den Tillaar et al. (2017) addressed this sprint specificity by comparing hamstring muscle activity and the hip and knee angle of peak activity during strengthening exercises and sprint. Their results showed that even if sprint and *Nordic-hamstring* exercise (NHE) had their hamstring muscle peak activity at a similar hip and knee angle, none of the isolated exercises (NHE and variations, standing-kick, lying-kick) that were tested induced hamstring electromyographic (EMG) activity >75% for the semimembranosus, 65% for the semitendinosus, and 40% for the biceps femoris of that measured during top-speed sprinting (van den Tillaar et al., 2017).

In addition, considering the role played by the hamstring muscle in producing force during sprinting (Morin et al., 2015), knowing how much strengthening exercises target horizontal force production capacity during sprinting could also be relevant in assessing the sprint specificity of exercises for both performance and preventative approaches. Such horizontal force production capacity can be assessed through the Force–velocity relationship during sprinting, summarized by its two extremums characterizing the horizontal force production capacity at low (F0) and high (V0) speed (Samozino et al., 2016; Morin et al., 2019).

To summarize, hamstring muscles play a major role in horizontally oriented force production during sprinting (Morin et al., 2015), but sprinting is a major cause of HMI (Edouard et al., 2016; Ekstrand et al., 2016). To date, little is known about the level of sprint-specificity of different isolated hamstring muscle-strengthening exercises (van den Tillaar et al., 2017), but this information could help identify which strengthening exercises solicit hamstring muscles in the closest sprinting modalities. A hamstring-strengthening exercise specific to sprinting would be an exercise that activates hamstrings significantly and that targets horizontal force production during sprinting. Besides primary and secondary injury prevention, such exercises would also be relevant in improving horizontal force production from a performance strategy perspective.

Therefore, the objectives of this study were: (1) to analyze the correlations between horizontal force production capacities during sprinting and the force production capacity during hamstring muscle-strengthening exercises, and (2) to compare hamstring muscle activity during sprinting and hamstring muscle-strengthening exercises. We first hypothesized that performance achieved during hamstring-dominant exercises executed in similar conditions to hamstring force production during sprinting would be correlated with the horizontal force production capacity during sprinting. Therefore, it could be expected that performance during exercises executed in full leg extension and high velocity would be correlated with horizontal force production at high running velocities (V0) and not at low running velocities (F0). On the opposite, performance in exercises executed at a low or null velocity, and without full leg extension, would be correlated with horizontal force production capacity at low running velocities (F0) and not at high running velocities (V0). Based on the results of van den Tillaar et al. (2017) during treadmill sprinting, the second hypothesis of this work was that the level of activity reached during over-ground sprinting cannot be reproduced with hamstring-strengthening exercises.

## Materials and Methods

### Study Design and Procedure

We conducted an experimental cross-sectional study with adult male and female sprinters, who performed, during one session, two maximal sprints and six exercises selected for their ability to target hamstring muscles. Hamstring force production and muscle activity were assessed during the maximal sprints and the exercises. The study protocol was reviewed and approved by the Saint-Etienne University Hospital Ethics Committee (Institutional Review Board: IORG0007394; IRBN322016/CHUSTE).

### Population

Participants were recruited from French athletics clubs and were included if they practiced sprinting, trained at least three times per week, were engaged in strength training on a weekly basis, and accepted to participate in this study. Participants with any recent history (<6 months) of HMI were excluded from the study, as were participants reporting pain or discomfort great enough to influence their abilities to sprint or to perform one of the exercises.

Sample size calculations (G^*^Power software, version 3.1.9.2, Germany) were determined to allow us (1) to analyze the correlations between horizontal force production capacities during sprinting and the force production capacity during hamstring muscle-strengthening exercises (to detect correlation of 0.7 between variables), and (2) to compare hamstring muscle activity during sprinting and hamstring muscle-strengthening exercises [based on the minimum difference reported in hamstring muscle activity between strengthening exercise and sprint (van den Tillaar et al., 2017)]. With a significance value of 0.05 and desired power of 0.80, a sample size of 13 subjects was calculated for this analysis.

### Experimental Protocol

Testing was performed during one session per participant at their training center, on different days for each athlete but with similar weather conditions. Participants were asked to bring their own clothes and running shoes with spikes. The testing lasted 1 h and consisted in baseline data collection, a warm-up, two maximal sprints, and six exercises, always following the same order. Anthropometric data (age, mass, and height) were collected at the beginning of the testing by a physiotherapist. Personal information regarding participants' level of sport practice, years of practice, training frequency, and present or past HMIs was collected through an online survey.

**Figure 1:**
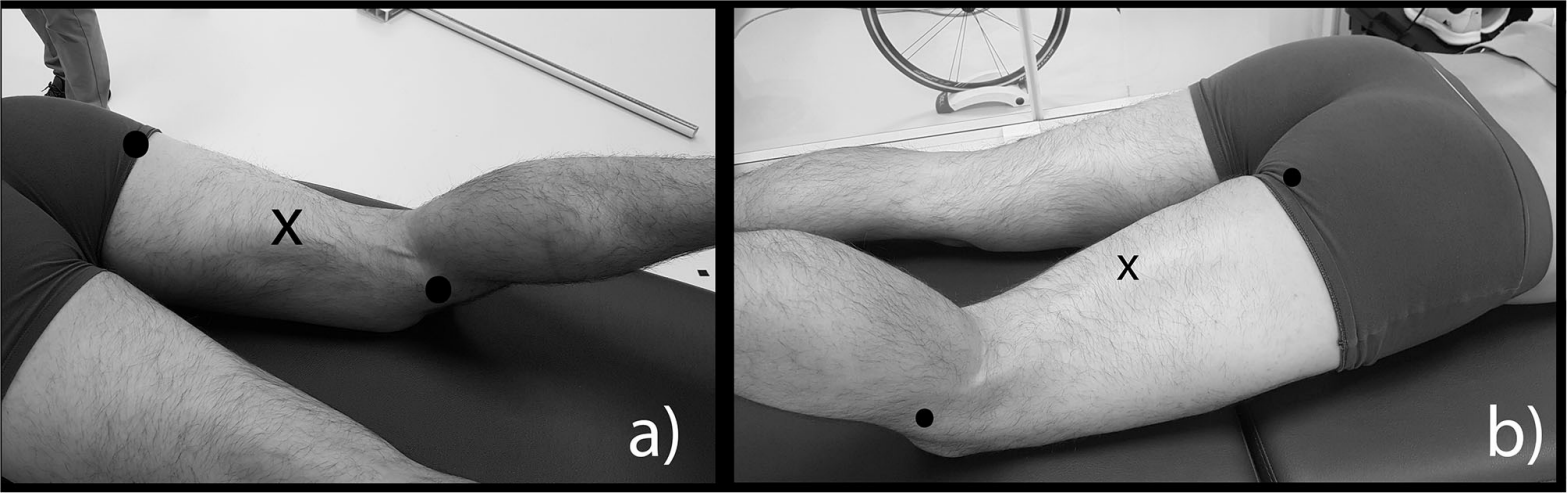
EMG electrodes placement for semitendinosus **(a)** and biceps femoris long head **(b)**.

Each participant had to perform a personalized 30 min warm-up, based on a similar structure and consisting in moderate running, specific athletics movements (e.g., drills), and accelerations. After the warm-up period, participants were allowed 5–10 min of free cool-down during which EMG surface electrodes (in bipolar configuration) were placed by a physiotherapist (CP) on the biceps femoris long head (BF) and the semitendinosus (ST) of the participant's dominant leg, on skin that had been shaved, abraded, and cleaned with alcohol. Electrode placement on the ST and BF muscles respected the presumed direction of the muscle fibers and SENIAM recommendations (Hermens et al., 2000) (Figure 1). The reference electrode was placed on the head of the fibula.

After the resting period of 15 min, each participant performed two maximal sprints over a 50 m distance. Sprints were performed on an outdoor synthetic running track with spiked shoes and starting from a crouched position. A resting period of 3–5 min was allowed between the two maximal sprints, and 10 min of rest after the two sprints and before the six exercises.

Then, each participant performed six exercises. These exercises were chosen based on previous studies (Bourne et al., 2017; van den Tillaar et al., 2017; Hegyi et al., 2019a) reporting exercises activating hamstring muscles significantly and targeting horizontal force production during sprinting, and based on their ability to target hamstring muscle in various modalities which could resemble those found during the different phases of the sprint. More specifically, the *upright hip extension* (UHE) was chosen for its ability to assess the hamstring muscle capabilities as horizontal force producers in a position closer to the stance phase of a sprint (i.e., standing and performed on one leg) (Malliaropoulos et al., 2012). The *standing kick* (SK) was chosen for its ability to assess indirect hamstring muscles eccentric capabilities at high velocity (van den Tillaar et al., 2017) and for its likeliness with late swing phase positioning. The *slide leg bridge (SB)*, as well as the *nordic hamstring exercise* (NHE), were chosen for their ability to bring high levels of hamstring muscles neuromuscular activity (Hegyi et al., 2019c) and for their ability to assess hamstring muscle eccentric capabilities at low speed (Bourne et al., 2017; Hegyi et al., 2019c). Details of placements, instructions given to the participants, and a full description of the exercises is provided in Table 1 and in the Supplementary Material. Exercises were performed three times after three trials of familiarization and in a random order to avoid potential fatigue bias. No familiarization session and no instructions were given regarding the exercise presentation before the testing.

**Table 1:** Details of placements, instructions, and measurements taken during the protocol.

	**Sprints** 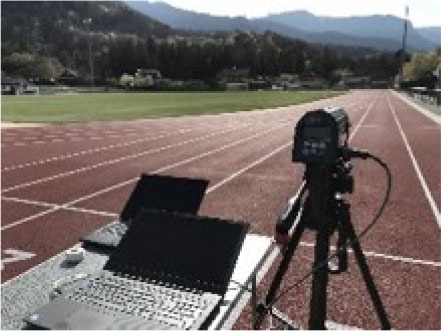	**Nordic Hamstring hip at 0^**°**^ (NHE0)** 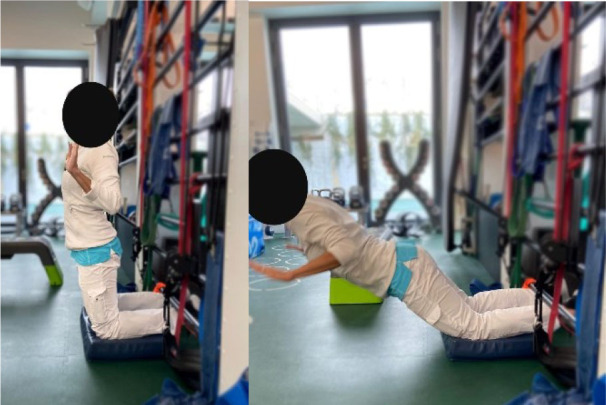	**Nordic hamstring hip at 90^**°**^ (NHE90)** 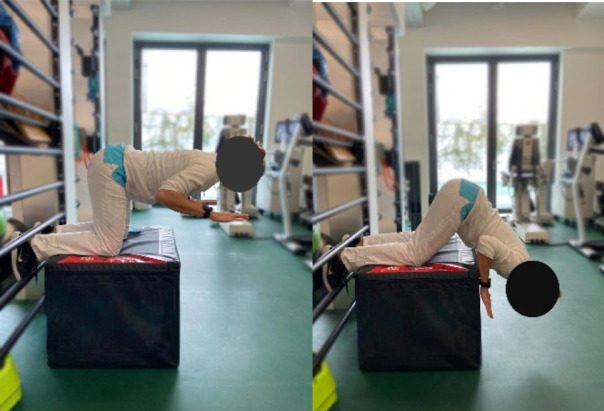	**Upright hip extension (UHE C/I)** 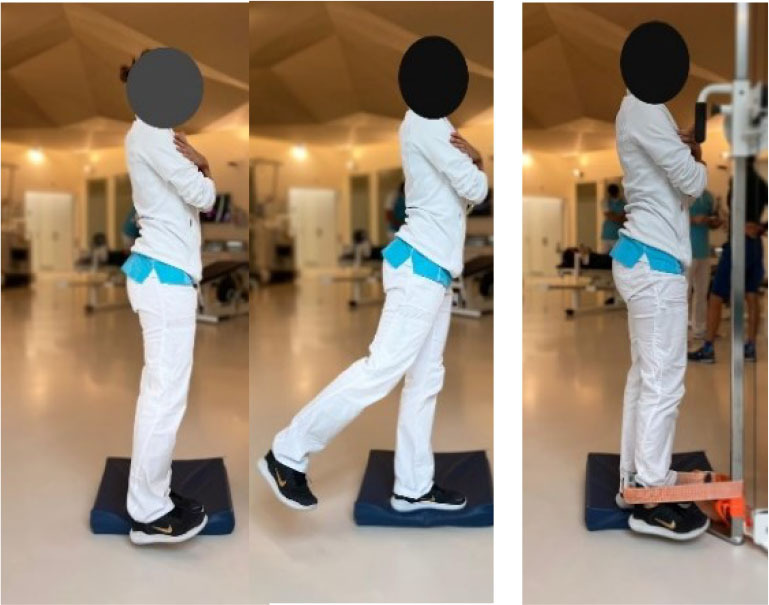	**Standing Kick (SK)** 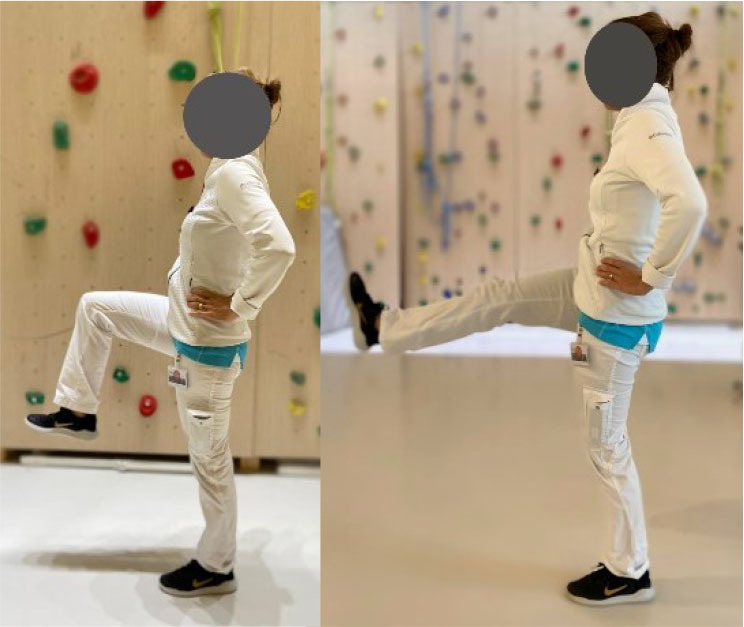	**Slide leg Bridge (SB)** 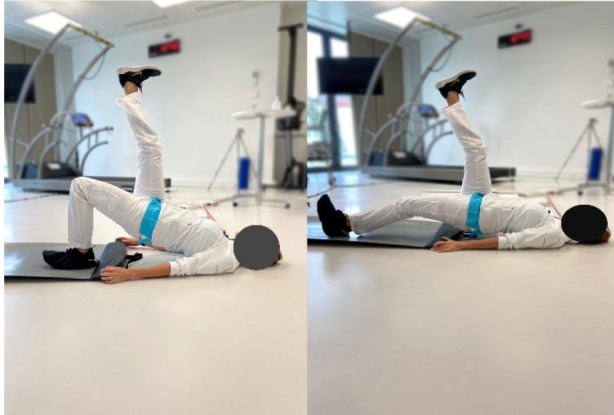
Variable of interest for hamstring performance assessment	Horizontal force production capacity at low (F0) and high (V0) velocities	Hamstring muscle eccentric force production capacity at low velocities with higher (NHE90) or lower (NHE0) muscle lengths		Hamstring muscle concentric force production capacity at low (UHE-I) and high (UHE-C) velocities standing with leg straight	Indirect Hamstring muscle eccentric force production capability in the same lower limb configuration as for the end swing of the leg	Low maximal speed as an index of hamstring muscle eccentric force production capability
Placement	The participant is placed on a crouched position behind the starting line. The subject is asked to sprint as fast as possible until the 50 meters line	The participant is positioned on a bench on his/her knees with feet self-fixed. The subject is asked to fall forward as slow and as long as possible and keep arms crossed and hips in a neutral position	The participant is positioned on a bench on his/her knees with feet self-fixed. The subject is asked to fall forward as slow and as long as possible and keep arms crossed and hips in a 90° position	The participant is standing on one foot with hands on shoulders. The subject is asked to do a hip extension as fast as possible while keeping the lower leg straight	The participant is standing on his/her non-dominant leg with hands crossed on shoulders. The dominant leg is bent (90° hip and knee flexion). The subject is asked to do a knee extension as fast as possible while keeping the hip flexed and without losing balance	The subject is positioned supine with the dominant leg semi-flexed with foot on the ground, the non-dominant leg is stretched with foot in the air. The subject is asked to let his foot slide down to stretch the knee as slowly as possible
Set point	“Run as fast as possible until you reach the 50 meters line”	“Let yourself fall forward gently, restraining yourself only with the strength of your thighs. When the tension on the back of your thighs is too great, let go and put your hands on the ground”	“Let yourself fall forward gently, restraining yourself only with the strength of your thighs. When the tension on the back of your thighs is too great, let go and put your hands on the ground”	“While keeping your hands on your shoulder and your leg straight, shift your weight on your non-dominant leg and do a hip extension as fast as possible with the dominant leg. You cannot bend forward”	“While staying on one leg and without losing balance, kick as fast as possible with your flexed leg”	“While keeping one leg up, slip the other foot as slowly as possible on the ground. Until you cannot maintain your weight”
Measured parameters	Instantaneous displacement	Break angle		Maximum velocity (UHE-C) or force (UHE-I)	Maximum velocity	
Tools used for parameters recording	Radar Stalker ATS©	Video recording, 240 fps, kinematic analysis software Kinovea ©		Linear encoder and LabVIEW© program		

### Data Collection and Analysis

#### EMG Analysis

The EMG signal of the ST and BF throughout the entire sprinting and exercises was measured at 1,000 Hz using a Data LOG MWX8 system (mass = 125 g; Biometrics Ltd., Newport, UK). The signals were rectified, smoothed (25 moving window), put through a lowpass band filter (Butterworth type, fourth order, and cut-off frequency at 8 Hz) and analyzed using the Analyze© software. The rectified, smoothed, and filtered signal was averaged over each burst. Since Hegyi et al. (2019b) recently reported greater EMG activity during sprints at a different speed than during maximal voluntary contraction, we used the greater EMG activity sustained over the two sprints to establish the maximal value (100%), and examined muscle activity during the various exercises in relation to that maximal value.

#### Sprint Analysis

Instantaneous velocity was recorded by using a Radar Stalker ATS II© system fixed on a tripod at 1 m height and placed 2 m behind the participants. A method based on a biomechanical model, anthropometric data (height and weight), and instantaneous velocity during a sprint acceleration was used to determine individual force–velocity relationships from linear regression (Samozino et al., 2016; Morin et al., 2019). Only the sprint part from the start to when the speed had reached its maximum plateau was considered. From the force–velocity relationship, two parameters—theoretical maximal horizontal force production at null velocity (F0, intercept of the force–velocity curve with the force axis) and theoretical maximal velocity when the horizontal force can be produced (V0, intercept of the force–velocity curve with the velocity axis)—were used to characterize horizontal force production capacity during sprinting at low and high velocity, respectively.

#### Analysis of Hamstring Muscle-Strengthening Exercises

The performance parameters, data collection, and analysis are presented for each exercise in Table 1 and with additional information provided in the Supplementary Material.

#### Statistical Analysis

The normality of all variables was tested using the Shapiro-Wilk normality test. The intra-trial reliability of each mechanical variable was calculated using the two maximal values from the testing session. We calculated the Intraclass correlation coefficient (ICC; Two-way mixed effects, absolute agreement, single rater/measurement based on Koo and Li, 2016, as well as the standard error of measure (SEM %).

To test the correlation between sprinting horizontal force production capacities (F0 and V0) and hamstring muscle performance during exercises, Pearson's correlation tests (r), or Spearman's correlation tests (r_s_) when distribution normality was not observed, were used. To assess the magnitude of the correlations, confidence limit intervals at 95% (95% CI) of the correlation coefficient were determined and interpreted using the Hopkin's threshold (Hopkins, 2000) (*r* = 1: perfect correlation; 1 ≥ r ≥ 0.9 nearly perfect; 0.9 ≥ r ≥ 0.7 very large; 0.7 ≥ r ≥ 0.5: large; 0.5 ≥ r ≥ 0.3: moderate; 0.3 ≥ r ≥ 0.1: small; 0.1 ≥ r: trivial). The level of statistical significance was set at *p* < 0.05.

To compare the hamstring muscle EMG activity observed during each exercise with the maximal EMG activity during the sprint (100%), one-sample Student *t*-tests were used, or non-parametric Wilcoxon tests when normality distribution was not observed. After adjusting for Type 1 error, the level of statistical significance was set at *p* ≤ 0.008.

Statistical analyses were performed using the Microsoft Excel (Office, Microsoft, 2017) and JASP (JASP Team software, Version 0.9.1.0, University of Amsterdam).

## Results

### Study Participants

A total of 14 sprinters (7 women and 7 men; age (mean ± SD): 19.5 ± 3.2 years; body mass: 64.9 ± 8.8 kg; height: 1.72 ± 0.11 m; years of practice: 10 ± 4.6; hours of training per week: 7.0 ± 1.1) trained for sprint running (season personal best: 13.53 ± 0.65 s for women, and 11.84 ± 0.98 s for men in 100-m sprints, and 27.71 ± 1.35 s for women and 24.45 ± 1.00 s for men in 200 m sprints; at the time of the experimentation), and who met the inclusion criteria, gave their written consent to participate in the study.

### Correlation Between Sprinting Horizontal Force Production Capacities (F0 and V0) and Force Production During Exercises

Descriptive statistics and reliability measures for sprinting and hamstring muscle exercise variables are presented in Table 2.

**Table 2:** Means, standard deviations, and reliability measures for sprint and exercises data.

**Mechanical variables**		**Mean ± SD**	**ICC**	**SEM%**
Sprint	F0 (N/kg)	7.1 ± 0.6	0.996	6.62
Sprint	V0 (m/s)	8.6 ± 0.6	0.999	3.67
Sprint	Vmax (m/s)	8.2 ± 0.5	0.999	3.53
NHE 0	Break angle (°)	38 ± 17.8	0.996	6.55
NHE 90	Break angle (°)	35 ± 12	0.994	7.73
UHE-I	Maximal Force (N/kg)	0.54 ± 0.15	0.885	6.96
UHE-C	Peak speed (m/s)	4.3 ± 0.94	0.979	14.5
SK	Peak speed (m/s)	4.4 ± 0.93	0.985	2.44
SB	Peak speed (m/s)	0.16 ± 0.09	0.878	19.5
	**Biceps femoris long head**		**Semitendinosus**	
**Peak EMG activity [Normalized to sprint (%)]**				
Sprint	100		100	
NHE 0	46.1 ± 20.3		57.8 ± 18.3	
NHE 90	29.7 ± 9.4		40.8 ± 7.9	
UHE-I	54.0 ± 27.0		53.7 ± 20.7	
UHE-C	48.4 ± 22.9		55.2 ± 22.3	
SK	27.4 ± 19.4		29.5 ± 16.0	
SB	34.28 ± 16.7		39.3 ± 13.4	

*EMG, Electromyography, normalized to maximal burst of activity during sprint; SD, Standard deviation; NHE0 and NHE90, Nordic hamstring exercise at 0and 90° of hip flexion; UHE-I and UHE-C, Upright-hip-extension in isometric and concentric modalities; SK, Standing kick; SB, Slide Leg Bridge; F0 and V0, Theoretical horizontal force production at low and high speed. ICC, Intraclass correlation coefficient; SEM, Standard error of measurement*.

Reliability was excellent for SK (ICC: 0.96–0.99; SEM: 2.44%); for NHE0 (ICC: 0.95–0.99; SEM: 6.55%); for NHE90 (ICC: 0.90–0.99; SEM: 7.73%); reliability was good for UHE-C (ICC: 0.86–0.98; SEM: 14.5%) for SB (ICC: 0.78–0.97; SEM: 19.5%); for UHE-I (ICC: 0.81–0.97; SEM: 6.96%); for Vmax (ICC: 0.89–0.98; SEM: 3.53%), and for V0 (ICC: 0.85–0.98; SEM: 3.67%), and reliability was moderate for F0 (ICC: 0.69–0.96; SEM: 6.62%).

Non-parametric tests were used owing to the non-normal distribution of the data. Large positive statistically significant correlations were found between V0 and maximal force during the UHE-I exercise [r_s_ = 0.56 (95% CI 0.454–0.665); *p* = 0.040] and between V0 and the break-angle during both NHE0 [r_s_ = 0.66 (95% CI 0.554–0.764); *p* = 0.012] and NHE90 [r_s_ =0.73 (95% CI 0.625–0.832); *p* = 0.003] (Table 3). Large positive statistically significant correlations were found between F0 and the maximal force during UHE-I [r_s_ = 0.60 (95% CI 0.494–0.705); *p* = 0.028] and between F0 and the break-point angle during the NHE0 [r_s_ = 0.59 (95% CI 0.484–0.695); *p* = 0.030] (Table 3). No significant correlation were found between V0 and peak-speed during the SK, SB, or UHE-C. Results for correlation analyses are presented in Table 3, of whom, for information, those not directly associated to our hypotheses (e.g., correlation between performance during strengthening exercises at low velocity and V0, or vice-versa).

**Table 3:** Results of correlation analysis between horizontal force production during sprinting and hamstring muscles performance at exercises.

	**Spearman r_**s**_ (correlation magnitude)**	***p***	**Lower and upper limits (95% CI) of the r_**s**_**
V0 - NHE0	0.66 (large)	* 0.012	0.55 (large)−0.76 (very large)
V0 - NHE 90	0.73 (very large)	** 0.003	0.62 (large)−0.83 (very large)
V0 - UHE-I	0.56 (large)	* 0.040	0.45 (moderate)−0.66 (large)
V0 - UHE-C	0.35 (moderate)	0.215	0.25 (small)−0.45 (moderate)
V0 - SK	−0.29 (small)	0.209	−0.39 (moderate)−0.18 (small)
V0 - SL	0.29 (small)	0.299	0.18 (small)−0.39 (moderate)
F0 - NHE0	0.59 (large)	* 0.030	0.48 (moderate)−0.69 (large)
F0 - NHE90	0.43 (moderate)	0.126	0.32 (moderate)−0.54 (large)
F0 - UHE-I	0.60 (large)	* 0.028	0.49 (moderate)−0.70 (very large)
F0 - UHE-C	0.26 (small)	0.374	0.15 (small)−0.36 (moderate)
F0 - SK	0.04 (trivial)	0.892	0.00 (trivial)−0.14 (small)
F0 - SB	0.09 (trivial)	0.765	0.00 (trivial)−0.19 (small)

r_s_: Spearman's correlation coefficient: p: level of significance of the correlation *p < 0.05, **p < 0.01; CI confidence intervals for the r_s_; EMG, Electromyography; NHE0 and NHE90, Nordic hamstring exercise at 0 and 90° of hip flexion; UHE-I and UHE-C, Upright-hip-extension in isometric and concentric modalities; SK, Standing kick; SB, Slide leg bridge; F0 and V0, Theoretical horizontal force production at low and high speed.

### Comparison of the Hamstring Muscle EMG Activity Observed During Each Exercise With the Maximal EMG Activity During the Sprint

**Figure 2:**
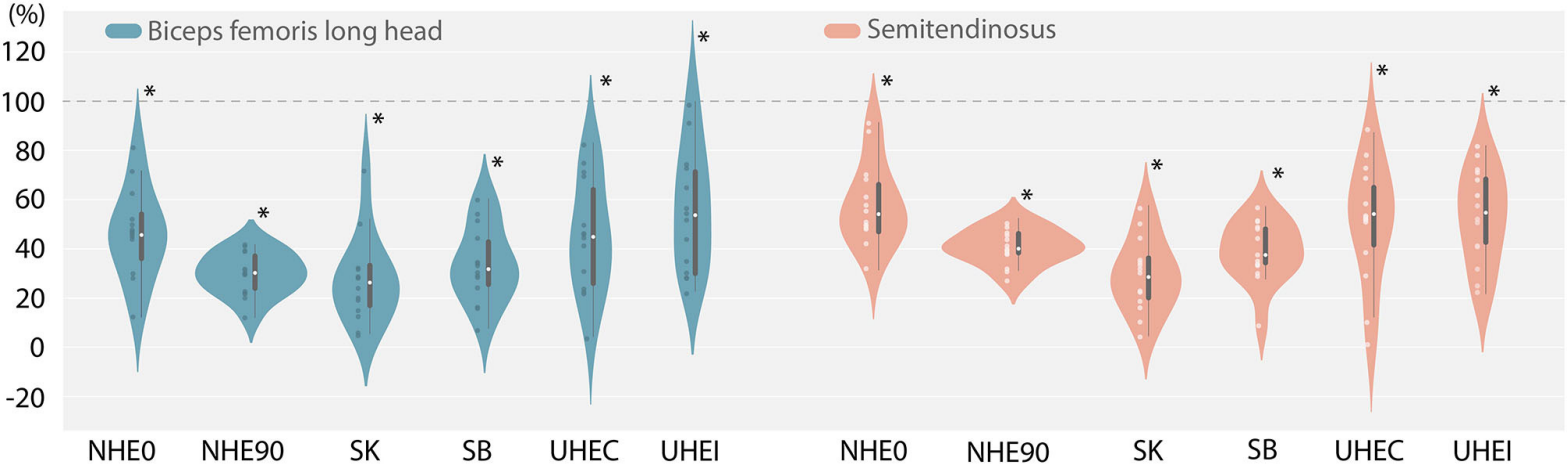
Individual distribution, median, quartiles, minimum, maximum of biceps femoris, and semitendinosus neuromuscular activity (EMG) during exercises (%) related to the maximal burst of activity during sprint (100%). *A significant difference between this EMG activity and 100% (*p* < 0.008). NHE0 and NHE90, Nordic hamstring exercise at 0 and 90° of hip flexion; UHE-I and UHE-C, Upright hip extension in isometric and concentric modalities; SK, Standing kick; SB, Slide Leg Bridge.

Parametric tests were used since all sets of EMG data followed a normal distribution. After a type-1 error adjustment, the one-sample Student *t*-test reported statistically significant differences (*p* < 0.008) between EMG activity during all exercises and the maximal EMG activity during the sprint (100%) (Figure 2).

## Discussion

The main findings of this study were that (1) performance during the *Nordic-hamstring* and the *Upright-Hip-Extension* in isometric (UHE-I) exercises were largely to very largely correlated with the force production during sprinting at both low (F0) and high (V0) velocity, and that (2) none of the exercises tested (NHE0, NHE90, UHE-I, UHE-C, SK, SB) induced hamstring EMG activity >60% (on average, for biceps femoris or semitendinosus) of the maximal hamstring EMG activity measured during maximal sprinting acceleration. This study is the first, to the authors' knowledge, to investigate the specificity of strengthening exercises in relation to determinants of over-ground sprinting mechanics in terms of force production functional capacity and surface EMG activity.

### Sprint-Specificity of Hamstring-Strengthening Exercises in Regard to Horizontal Force-Production During Sprinting

Our results show that the ability to reach a greater break-point angle (indirectly estimating eccentric force production capacity) during both NHE0 and NHE90 exercises was correlated with greater levels of force production at low and high speed (F0 and V0). These results are partially in agreement with our hypothesis, since we expected low-speed exercises (NHE) to be correlated with force production at low velocity, but not with high velocity. This could be interpreted as the fact that the functional capacity of the hamstring muscles in the eccentric modality may be related to the ability to produce backward-oriented force during the entire sprint acceleration. This highlights a possible relationship between sprint performance and the eccentric contraction modality of the hamstring muscles, and offers indirect support to Chapman and Caldwell's (Chapman and Caldwell, 1983) theory stating that the capability of the hamstring muscles to reduce the kinetic energy of the lower limb in the late swing (time when hamstring eccentric contraction has been suggested during sprint; Kenneally-Dabrowski et al., 2019) could be a determining factor in sprint performance, notably at high speeds (Chapman and Caldwell, 1983). Regarding the link between hamstring eccentric strength as assessed during the NHE and sprint mechanics or performance, results from the recent literature tend to be controversial (Ishøi et al., 2018; Markovic et al., 2019; Suarez-Arrones et al., 2019). Authors have reported benefits (Ishøi et al., 2018), no effect (Suarez-Arrones et al., 2019), or negative effects (Markovic et al., 2019) of NHE on sprint performance. Our results suggest a positive relationship between eccentric knee flexor strength and horizontal force production. However, interventional studies on track and field athletes are still needed to analyze the potential effect of NHE as an isolated exercise on sprint performance and/or HMI reduction.

Our results also revealed that the force production during the *Upright-Hip-Extension* in isometric (UHE-I) was strongly correlated with sprinting force production capacity at high (V0) and low velocity (F0). This suggests that hamstring force production at null velocity but maximal force is linked to horizontal force production capacities at both low- and high-speed running. As the UHE-I mimics the early stance phase, these results could support the findings of Clark and Weyand (2014) who reported that the impulse generated during the first half of stance is of great importance for sprint performance. Additionally, to date, most of the HMI prevention publications recommend that exercises target eccentric force strengthening (Bourne et al., 2018; van Dyk et al., 2019), and other strengthening modalities are promoted less. Our present results highlight the specificity of isometric strength capabilities with force production during sprinting and indirectly support the potential benefits of varying strengthening modalities, including isometric, as well as hip and knee position for exercises, in performance and HMI reduction (Kenneally-Dabrowski et al., 2019). However, these correlations were not directly associated to the initial hypotheses and may suffer from a slightly inflated type I error, which should be considered when interpreting the results.

### Hamstring Muscle Activity During Strengthening Exercises

We reported significantly higher hamstring muscle activity during maximal over-ground sprints than during the hamstring-specific exercises. Our results show a great inter-individual variability and some subjects reach levels of muscle activity close to the sprint, however considering the average of the subjects none of the exercises tested here activated hamstrings as much as 58% (for biceps femoris or semitendinosus) of what it maximally sustained during a sprint. These results were similar to those reported by van den Tillaar et al. (2017) from their study performed on treadmill sprinting. Therefore, our results suggest that sprinting would be the best exercise to highly activate the hamstring and to induce muscle adaptation. This supports the hypothesis of Edouard et al. (2019) stipulating that sprinting could be a relevant “exercise” in the sprint-related HMI management.

The highest hamstring muscle activity was recorded during exercises mimicking the early stance [*Upright-Hip-Extension* in isometric (UHE-I)] and stance [*Upright-Hip-Extension* in concentric (UHE-C)] phases of the sprint strikes. Similarly, Yu et al. (2008) reported maximal hamstring activity during the late swing phase and the early stance phase of an over-ground sprint. Regarding the higher EMG levels during the UHE-C, an explanation could be that this exercise solicits the hamstring on an open kinetic chain, which has been previously reported as increasing hamstring muscle activity (Andersen et al., 2006; Malliaropoulos et al., 2015).

Similarly to previous studies (McAllister et al., 2014; Bourne et al., 2017), higher hamstring muscle activity was reported during exercises in isometric or concentric modalities than in eccentric modalities. Additionally, from all the “eccentric exercises” tested, the *Nordic-hamstring* exercise performed at 0° of hip flexion (NHE0) allowed for greater hamstring muscle activity, which was also reported by previous studies (Bourne et al., 2017; van den Tillaar et al., 2017). In line with Hegyi et al. (2019c), the additional 90° of hip flexion during the *Nordic-hamstring* exercise (NHE90) did not facilitate greater hamstring muscle activity than the NHE0. To be more specific to sprint, Guex and Millet (2013) suggested to increase hamstring strain during exercises by increasing hip flexion. In our study, however, the hamstring muscle activity levels did not seem to increase from NHE90 to NHE0 (i.e., increasing hamstring strain). Controversially, exercises performed at negative strain (UHE-C) made it possible to achieve high levels of hamstring muscle activity while higher-strain exercises (*Standing-Kick* or *Nordic-hamstring* exercises) allowed for less muscle activity.

As EMG activity is correlated with the muscle force (Disselhorst-Klug et al., 2009), the low levels of EMG activity during the exercises could be explained by the sub-maximal intensities reached compared with sprint performed at maximal intensity. Comparing EMG activity during sprint with exercises performed at high and controlled intensities [such as the *Romanian-dead-lift* (Andersen et al., 2006; McAllister et al., 2014)] could give further credit to these exercises in terms of their specificity to sprint.

Although our results showed that the *Nordic-hamstring* exercise performed at 90° of hip flexion was specific to sprint from a force production standpoint, our study also revealed that hamstring muscle activity during this exercise was significantly lower than during sprint. To date, the *Nordic-hamstring* exercise is the most widely studied exercise for injury prevention (Gabbe et al., 2006; Bourne et al., 2018; van Dyk et al., 2019), and appears to be a key exercise in HMI reduction (Goode et al., 2015; Al Attar et al., 2017; van Dyk et al., 2019). Although van Dyk et al. (2019) reported its efficacy in decreasing HMI rates, it is not yet known whether this was due to performing a hamstring-strengthening exercise in eccentric modality, or the *Nordic-hamstring* exercises *per se*. Furthermore, since no studies have analyzed the efficacy of other exercises in preventing HMI, all of these pieces of information remain hypotheses only.

Finally, some of the exercises we tested were specific to sprint in terms of force production; however, the levels of hamstring muscle activity were heterogeneous across the various strengthening exercises and none of them could activate the hamstring as much as sprint could. Therefore, we think that using only one parameter (force production capabilities or levels of muscular activity) to explain sprint specificity could be a reductionist approach. In practice, our results highlight the fact that sprinting targets hamstring muscle in an irreplicable manner and should be part of any hamstring conditioning or injury prevention program.

### Methodological Considerations

Muscle hamstring performance was assessed via peak velocity during some strengthening exercises instead of measuring direct force production during the exercises. Given that the movement velocity depends on the force produced during this kind of exercise starting from null velocity, we chose to assess force production capabilities indirectly by measuring peak velocity. However, other parameters to address force production directly or indirectly could have been included. Regarding the *Nordic-hamstring* exercise, Sconce et al. (2015) reported a correlation between a greater “break-point angle” reached and a greater eccentric knee flexor torque. However, it appears that the angle of peak torque and the break-point angle are not correlated (Sconce et al., 2015). Additionally, the joint angles and velocities at which peak torque occurs during the *Nordic-hamstring* exercise might not be a reliable measure and greater eccentric force production might be recorded after the break-point angle is reached (Muggleton et al., 2015).

Considering the EMG normalization procedure, hamstring EMG activity during the isolated exercises was intrinsically linked to the sprinting exercises, therefore we could not compare BF with ST muscular activity during exercises nor establish the BF/ST ratio. Additionally, the use of single surface electrodes gives an estimation of only one area of the muscle (Hermens et al., 2000). Using more accurate electrodes such as high definition EMG (HDEMG) in our study could have provided more information, as these electrodes would give a larger spectrum of the muscle activity. Future studies could focus on this last point in order to clarify the specificity of hamstring strengthening exercises aiming to target the different sprint phases. Finally, the athletes tested here were intermediate level sprinters and the findings should be further confirmed on higher level ones.

## Conclusion

Knowing which isolated strengthening exercises solicit hamstring muscles in the closest functional modalities (in terms of force and EMG outputs) compared to sprinting is of great interest from a performance, primary prevention, and return-to-sport standpoint. The sprint-specificity of exercises assessed through force production and muscular activity revealed that force production ability during certain exercises such as the Nordic-hamstring or the Upright-Hip-Extension exercise were related to horizontal force production during sprinting. However, with none of these exercises was it possible to reach similar levels of EMG activity as the ones induced by sprinting activities. Maximum sprinting activities appears to be the only way to achieve high muscle activity. However, more investigation is needed to determine which exercise or combination of exercises could reduce hamstring injuries or increase sprint performance.

## Data Availability Statement

The raw data supporting the conclusions of this article will be made available by the authors, without undue reservation.

## Ethics Statement

The studies involving human participants were reviewed and approved by Saint-Etienne University Hospital Ethics Committee, Institutional Review Board: IORG0007394; IRBN322016/CHUSTE. Written informed consent to participate in this study was provided by the participants' legal guardian/next of kin.

## Author Contributions

CP collected the data and wrote manuscript. CP, PE, and PS designed the study and analyzed the data. CP, PE, PS, J-BM, JL, JM, and KG revised manuscript. All authors contributed to the article and approved the submitted version.

## Conflict of Interest

The authors declare that the research was conducted in the absence of any commercial or financial relationships that could be construed as a potential conflict of interest.
