# Supplementary material for: Sprint Specificity of Isolated Hamstring-Strengthening Exercises in Terms of Muscle Activity and Force Production
Source: Front Sports Act Living. 2021 Jan 21;2:609636. doi: 10.3389/fspor.2020.609636 (PMC7859261; doi:10.3389/fspor.2020.609636)
Supplement: Supplementary file 1 [file Data_Sheet_1.pdf]

Supplementary material for the manuscript entitled: Sprint-specificity of isolated hamstring strengthening exercises in terms of muscle activity and force production

Data acquisition and analysis for the Nordic hamstring exercise.

The *nordic hamstring exercise* (NHE0) was chosen for its ability to assess hamstring muscle eccentric capabilities at low speed (Brockett, Morgan, & Proske, 2001). During the NHE0, participants were placed on a 45 cm height bench, on their knees with their feet maintained by the bench. Participants were asked to let themselves fall forward as slowly and for as long as possible using their hamstring muscles, and to try keeping tension in their hamstring muscles while keeping their hips fixed in a fully extended position throughout the whole range of motion. The classical NHE0 is performed at relatively short muscle length, with negative to null strain stress on the hamstring (varying from -90° to 0°) (Guex & Millet, 2013). Therefore, the NHE90 was chosen as well as it places hamstrings in relatively longer length (Hegyi et al., 2019). During the NHE90, participants were placed on the same bench on their knees with their feet self-maintained by the bench. Participants were asked to bend their hips at a 90° angle and to let themselves fall forward as slowly and for as long as possible using their hamstring muscles, and to try keeping tension in their hamstring muscles while keeping their hips fixed at a 90° angle position throughout the whole range of motion. The strain stress on the hamstring varying from 0° to 90°. The break point angle was considered as an index of hamstring muscle eccentric force production capacity (the higher the break angle, the higher the eccentric force production capacity) (Lee, Li, Yung, & Chan, 2017). The performance parameter recorded during this exercise was the “break point angle” (Lee, Li, Yung, & Chan, 2017) corresponding to the angle of knee flexion at which participants could not hold the tension and had to “let go”. As suggested in the literature (Lee et al., 2017), the break angle is the angle were angular velocity reached more than 10°/s. This

angle appears to be correlated to knee-flexors eccentric peak torque. Both exercises (NHE0 and NHE90) were video recorded to have access to linear and angular speed and then extract the position data. The video was recorded using an IPad© (in slow motion mode, allowing a 240fps) and instantaneous displacement was extracted using the Kinovea© kinematic software, datas were smoothed (20 moving window) and filtered (Butterworth fourth order) before being transformed into linear (derivation over time) and angular (linear velocity divided by the thigh length) variables using Microsoft Excel software.

Data acquisition and analysis for the upright hip extension exercise

The *upright hip extension* (UHE) was chosen for its ability to assess the hamstring muscle capabilities as horizontal force producers in a position closer to the stance phase of a sprint (i.e. standing and performed on one leg) (Malliaropoulos et al., 2012). To assess hamstring muscle force production at different speed modalities, the UHE was perform in isometric (UHE-I) and concentric (UHE-C). During the UHE-I exercise, the participants were standing against a wall on their non-dominant leg. Participants were asked to push as strongly as possible into the wall with their dominant leg while keeping their arms crossed on their shoulders. The strain stress on the hamstring was null for the UHE-I and varying from 0° to -30°. This maximal force was considered as an index of hamstring muscle concentric force production capability at low velocity. During the UHE-C exercise, the participants were standing on their non-dominant leg with hands crossed on shoulders and were asked to do a hip extension as fast as possible with their dominant leg while keeping the lower leg straight. The strain stress on the hamstring was varying from 0° to -30. The maximal horizontal velocity reached by the heel was used as an indirect index of the hamstring muscle concentric force production capability. For the isometric modality (UHE-I), the performance parameter recorded was the maximal force

produced during a single push, using a handheld dynamometer (Micro FET2©) placed behind the participant's heel and against the wall. Raw force data were normalised to body mass. For the concentric modality (UHE-C), the force production was indirectly assessed through the peak velocity reached during the movement, considering that the higher the force, the higher the foot acceleration, the higher the peak velocity (principles of Dynamics). This maximal velocity was considered as an index of hamstring muscle concentric force production capability of hip extensors at high velocity. The maximal velocity was derived from the position changes of the heel's participants. Position changes were recorded at 100 Hz with the use of a linear encoder (Kübler Group, Villingen-Schwenningen, Germany) fixed in front of the participants and with a cable attached to the heel's participants and connected to a National Instrument© Data Acquisition Card (NI USB 6009) and controlled by a self-made LabVIEW© program conceived for this study. The signals were smoothed (20 moving window) and filtered (Butterworth fourth order) before obtaining maximal speed out of the three individual trials.

Data acquisition and analysis for the standing kick exercise

The *standing kick* (SK) was chosen for its ability to assess indirect hamstring muscles eccentric capabilities at high velocity (van den Tillaar et al., 2017) and for its likeliness with late swing phase positioning. The maximal horizontal velocity reached by the heel was used as an indirect index of the hamstring muscle eccentric force production capability to counterbalance the quadriceps action inducing the swing of the leg. During the SK test, participants were standing on their non-dominant leg with dominant leg hip at 90° flexion, knee at 90° flexion, and arms crossed on their shoulders. They were then asked to do a leg extension as fast as possible, without moving the hip angle or losing

balance. The strain stress on the hamstring was varying from 0° to 90° during this exercise. The performance parameter recorded was the maximal speed, recorded with a linear encoder (self-made linear encoder connected to a National Instrument© Data Acquisition Card and controlled by a LabVIEW© program) fixed behind the participants at a 50 cm height (to be horizontally aligned with the heel at the starting position) and with the cable attached to the heel's participants.

Data acquisition and analysis for the slide leg bridge exercise

The *slide leg bridge (SB)*, was chosen for its common use in hamstring muscle strengthening training (Malliaropoulos et al., 2012) and for its ability to bring high levels of hamstrings neuromuscular activity (Tsaklis et al., 2015). During the SB test, participants were positioned supine with the dominant leg semi-flexed with foot on the ground, the non-dominant leg was stretched with the foot in the air. The subject was asked to let his foot slide down to stretch the knee as slowly and as longer as possible until the leg was fully extended. To maximize gliding, a slide-board© (a 2 meter long mat with gliding surface) was used. The strain stress on the hamstring was varying from -90° to 0° during this exercise. Low maximal speed was an index of high hamstring muscle eccentric force production capacity. The performance parameter was maximal speed, recorded with a linear encoder (Kübler Group, Villingen-Schwenningen, Germany) fixed horizontally at the participant's feet and with the cable attached to the heel's participants. The encoder was connected to a National Instrument© Data Acquisition Card and controlled by a LabVIEW© program.

References

Brockett, C., Morgan, D., & Proske, U. (2001). Human hamstring muscles adapt to eccentric exercise by changing optimum length. *Medicine and Science in Sports and Exercise*, *33*(5), 783–790. Retrieved from http://insights.ovid.com

Guex, K., & Millet, G. P. (2013). Conceptual Framework for Strengthening Exercises to Prevent Hamstring Strains. *Sports Medicine*, *43*(12), 1207–1215. https://doi.org/10.1007/s40279-013-0097-y

Hegyi, A., Lahti, J., Giacomo, J.-P., Gerus, P., Cronin, N. J., & Morin, J.-B. (2019). Impact of Hip Flexion Angle on Unilateral and Bilateral Nordic Hamstring Exercise Torque and High-Density Electromyography Activity. *Journal of Orthopaedic & Sports Physical Therapy*, 1–37. https://doi.org/10.2519/jospt.2019.8801

Lee, J. W. Y., Li, C., Yung, P. S. H., & Chan, K. M. (2017). The reliability and validity of a video-based method for assessing hamstring strength in football players. *Journal of Exercise Science and Fitness*, *15*(1), 18–21. https://doi.org/10.1016/j.jesf.2017.04.001

Malliaropoulos, N., Mendiguchia, J., Pehlivanidis, H., Papadopoulou, S., Valle, X., Malliaras, P., & Maffulli, N. (2012). Hamstring exercises for track and field athletes: injury and exercise biomechanics, and possible implications for exercise selection and primary prevention. *British Journal of Sports Medicine; London*, *46*(12), 846. https://doi.org/http://dx.doi.org.ezproxy.ulb.ac.be/10.1136/bjsports-2011-090474

Tsaklis, P., Malliaropoulos, N., Mendiguchia, J., Korakakis, V., Tsapralis, K., Pyne, D., & Malliaras, P. (2015). Muscle and intensity based hamstring exercise classification in elite female track and field athletes: implications for exercise

selection during rehabilitation. *Open Access Journal of Sports Medicine*, *6*, 209–217. https://doi.org/10.2147/OAJSM.S79189

van den Tillaar, R., Solheim, J. A. B., & Bencke, J. (2017). Comparision of hamstring muscle activation during high-speed running and various hamstring strengthening exercises. *International Journal of Sports Physical Therapy*, *12*(5), 718–727. https://doi.org/10.26603/ijspt20170718
